# A Household-Based Cross-Sectional Survey of Knowledge, Awareness and Practice Regarding Malaria in Western Area Rural District, Sierra Leone

**DOI:** 10.3389/fpubh.2021.664971

**Published:** 2021-03-18

**Authors:** Lili Wang, Jianhai Yin, Canjun Zheng, Samuel Juana Smith, Esther Ngegba, Xiaoxia Huang, Anitta Kamara, Xia Chen, Xu Wang, Wei Luo, Biao Kan

**Affiliations:** ^1^Center for Global Public Health, Chinese Center for Disease Control and Prevention, Beijing, China; ^2^National Institute of Parasitic Diseases, Chinese Center for Disease Control and Prevention, Chinese Center for Tropical Diseases Research, Shanghai, China; ^3^Division of Infectious Diseases, Chinese Center for Disease Control and Prevention, Beijing, China; ^4^National Malaria Control Program, Ministry of Health and Sanitation, Freetown, Sierra Leone; ^5^National Institute for Viral Disease Control and Prevention, Chinese Center for Disease Control and Prevention, Beijing, China; ^6^National Institute for Communicable Disease Control and Prevention, Chinese Center for Disease Control and Prevention, Beijing, China; ^7^National Center for AIDS/STD Control and Prevention, Chinese Center for Disease Control and Prevention, Beijing, China

**Keywords:** malaria, KAP survey, Western Area Rural District, Sierra Leone, national malaria strategic plan

## Abstract

Sierra Leone is a highly endemic area for malaria, and the implementation of the National Malaria Strategic Plan (2016–2020) has reached its midpoint in 2018. To provide more specific guidance for interventions in the future, a household-based cross-sectional survey was conducted to elucidate the knowledge, awareness and practices regarding malaria and malarial control measures among the general public. Three communities (Grafton, Jui, and Kossoh) in the Western Area Rural District that were in close proximity to Sierra Leone's capital city of Freetown were included. Households were randomly selected and interviewed with a structured questionnaire covering malaria infection, diagnosis, treatment and prevention, as well as knowledge of malaria prevention. As a result, a total of 262 qualified questionnaires were included. The average cost for meals per day is ~30,000 Leones in each household. The rate of awareness, indicated by reporting having heard of malaria, was 98.1% (257/262), and 86.6% (227/262) of the respondents knew that mosquito bites are the main route of transmission. In addition, 80.9% (212/262) of the respondents sought health advice or treatment for the illness, and a similar percentage of respondents had been tested for malaria, mostly with rapid diagnostic tests (RDTs). A high demand for long-lasting insecticidal nets (72.1%) matched the serious shortage (61.8%, 162/262), and of the households that reported a lack of nets, 66 had children younger than 5 years old. In conclusion, public awareness of malaria prevention is high, based on this survey, although there was a limited use of preventive measures in these three communities and the malaria burden was still high. Therefore, the public's knowledge of malaria should be sustained and reinforced, and the distribution and use of malaria prevention measures should be promoted to supprt the achievement of the planned objectives.

## Introduction

Malaria is endemic in Sierra Leone; the entire population is at risk of exposure because Sierra Leone is an area of stable malarial endemicity, and almost cases were infected with *Plasmodium falciparum* (1). Sierra Leone's high malaria disease burden accounted for ~48% of outpatient morbidity and ~38% of mortality in children younger than 5 years according to the national Malaria Indicator Survey conducted in 2016 (2). Although significant progress with regard to reducing mortality has been made, it is still a major public health concern. The control of malaria remains a priority in the national health agenda of Sierra Leone.

A multipronged strategy to control malaria, including vector control interventions, diagnostic testing, treatment with quality-assured artemisinin-based combination therapies, and standardized case management training manuals for community and facility treatment providers, has been developed and implemented in Sierra Leone based on the World Health Organization (WHO) recommendations (3). The goals are for 80% of the population to have appropriate levels of knowledge and uptake/practice of malaria prevention and management strategies, all the at-risk population to have access to the appropriate preventive interventions, 80% of individuals with suspected malaria cases to have access to confirmatory diagnostic testing, and all individuals with malaria to receive effective treatment by 2018, according to the current Sierra Leone National Malaria Strategic Plan (2016–2020) (SL-NMSP) (3).

The awareness of malaria symptoms, transmission, prevention and treatments is closely related to the implementations of malaria control programs (4–6). A knowledge, awareness and practices (KAP) survey about malaria could help policy makers better understand the current situation and guide malaria control strategies. Therefore, this study is conducted to determine the knowledge, awareness and practices regarding malaria and its control measures in the general public through a household-based survey at the midpoint of the implementation of the SL-NMSP.

## Materials and Methods

### Study Site

Three communities (Grafton, Jui, and Kossoh) in Western Area Rural District within close proximity to Sierra Leone's capital city Freetown were included in this household-based survey on malaria. This district is home to a very religiously and ethnically diverse population. Grafton is a trade center. Jui and Kossoh are neighborhood towns. Kossoh town is surrounded by a large forest reserve, and its major industries are farming and coal mining.

### Participants, Questionnaire, and Data Collection

A total of 300 households in the three designated communities were randomly selected as survey respondents using a cluster sampling design. One adult member from each selected household was interviewed with a structured questionnaire covering demographic information, economic status, basic knowledge of malaria, and malaria prevention and control practices. When medicines were discussed, packages of various drugs were displayed to the participants.

The survey was conducted in collaboration with the National Malaria Control Program of the Ministry of Health and Sanitation, Sierra Leone, in December 2018, at the midpoint of the SL-NMSP (3). Local staff from Sierra Leone-China Friendship Biological Safety Laboratory (7) were trained in prior to their involvement in the administration of the survey.

### Data Analysis

Data was input and cross-checked by EpiData version 3.1 (8, 9). Then, descriptive statistics and percentages were presented using Microsoft Excel 2010 software. Differences in distribution among three communities were evaluated using the chi-square (χ^2^) test or Fisher's exact test by SPSS software version 20.0 (IBM, USA) and *P* < 0.05 was considered statistically significant.

## Results

A total of 300 households were investigated, and 262 qualified questionnaires from 108, 47, and 107 households in Grafton, Jui, and Kossoh communities, respectively, were finally included in the analysis after checking for questionnaire completion and logic. The survey covered 1,645 residents, with ~6 persons per family. The average cost of meals per day was ~30,000 Leones in each household (one US dollar was equal to 8,000 Leones in December 2018).

### Malaria Infection, Diagnosis, and Treatment

A total of 929 persons in 202 households reported having had a fever in 2018. Among them, 406 persons from 97 households, 228 persons from 46 households, and 295 persons from 89 households in Grafton, Jui, and Kossoh communities, respectively, reported having suffered malaria. Moreover, 129 persons from 64 households reported having suffered malaria once, and 146 persons from 66 households reported having suffered malaria being twice, and 61 persons from 26 households being three times, and 275 persons from 91 households being more than three times, respectively.

Moreover, ~80.9% (212/262) of the respondents reported seeking professional advice or treatment for the illness from any source (*P* < 0.001, Fisher's exact test) (Table 1). Additionally, 72.2% (153/212) of the respondents reported preferring to seek advice and treatment from the public medical sector (including government hospital, government health center, mobile clinic, community health worker, other public sector) only (*P* = 0.001, Fisher's exact test). And 20.3% (43/212) and 7.1% (15/212) were found to seek assistance from the private sector (including private hospital, private clinic, mission/faith-based hospital, mission/faith-based clinic, pharmacy, mobile clinic, other private medical sector) only or any sector (Tables 1, 2).

**Table 1:** The distribution of medical sectors people seeking health advice or treatment in the three communities in the Western Area Rural District, Sierra Leone, 2018.

**Medical sector**	**Community**			**Total**
	**Grafton**	**Jui**	**Kossoh**	
Public sector only	68	29	56	153
Private medical sector only	15	5	23	43
Both sectors	2	10	3	15
Don't answer	1	0	0	1
Total	86	44	82	212

**Table 2:** The detail of public or private medical sectors people selected for health care seeking in the three communities in the Western Area Rural District, Sierra Leone, 2018.

	**Public medical sectors**						**Private medical sectors**							
**Community**	**Government hospital**	**Government health center**	**Mobile clinic**	**Community health worker**	**Other public sector**	**Subtotal**	**Private hospital**	**Private clinic**	**Mission/Faith-based hospital**	**Mission/faith-based clinic**	**Pharmacy**	**Mobile clinic**	**Other private medical sector**	**Subtotal**
Grafton	38	14	11	3	4	70	9	0	0	0	1	5	2	17
Jui	37	1	1	0	0	39	2^*^	1	1	10^*^	2	0	0	16
Kossoh	40	12	2	3	2	59	14^*^	0	0	6^*^	7	0	0	27

* *There was one respondent seeking advice or treatment both in the private sectors of private hospital and Mission/Faith-based Clinic in each community*.

Furthermore, 84.4% (221/262) of the respondents reported that they and their family members always visited a health care worker or doctor when they suspected they had contracted malaria (*P* = 0.071, Fisher's exact test). Among the individuals who went to see a health care worker or doctor, they reported visiting a professional every time (43.4%, 96/221) or most of the times (37.1%, 82/221) they suspected they had contracted malaria (χ^2^ = 37.219, *P* < 0.000) (Table 3).

**Table 3:** Malaria diagnosis and anti-malarial medicine taking in the three communities in the Western Area Rural District, Sierra Leone, 2018.

**Question and answer**	**Community**			**Total**	**Percentage**
	**Grafton**	**Jui**	**Kossoh**		
**1. When you and your family suspected you were infected with malaria, did you always go to see a health worker/doctor?**					
Yes	92	40	89	221	84.4%
No	12	1	7	20	7.6%
Don't answer	4	6	11	21	8.0%
**1.1 How often?**					
Every time	45	6	45	96	43.4%
Most of time	33	29	20	82	37.1%
Seldom	11	4	11	26	11.8%
Only when they showed severe condition	3	1	13	17	7.7%
**2. When you or any member of your family suspected that they had malaria, did the health worker/doctor conduct a malaria test?**					
Yes	88	44	78	210	80.2%
No	16	2	13	31	11.8%
Don't answer	4	1	16	21	8.0%
**3. Did the Doctor/health worker explain to you the test to be done?**					
Yes	64	25	55	144	55.0%
No	27	4	26	57	21.8%
Don't remember	9	17	5	31	11.8%
Don't answer	8	1	21	30	11.5%
**4. What type of test did they say they were going to perform?**					
RDT	57	17	69	143	54.6%
Microscopy	2	1	2	5	1.9%
Don't remember	41	27	8	76	29.0%
Don't answer	8	2	28	38	14.5%
**5. When you and your family were infected with malaria, did you take the anti-malarial medicine?**					
Every time	54	6	48	108	41.2%
Most of time	37	37	20	94	35.9%
Seldom	12	3	11	26	9.9%
Never	1	0	1	2	0.8%
Only when they showed severe condition	0	0	15	15	5.7%
Don't answer	4	1	12	17	6.5%
**6. What anti-malarial medicines did you take?**					
Sp/Fansidar	4	2^*^	0	6	2.3%
Combination with Artemisinin	2	1	0	3	1.1%
Artesunate+Lumafantrine	58^#^^,^^&^^,^^@^	32^*^^,^^$^^,^^%^	61^#^	151	57.6%
Artesunate+Amodiaquine	19^#^^,^^@^	14	21^#^	54	20.6%
Amodiaquine	6^Φ^	2^$^	1	9	3.4%
Quinine	0	1^%^	1	2	0.8%
Others: specify	29^&^^,^^@^^,^^Φ^	0	10	39	14.9%
Don't answer	2	0	16	18	6.9%
**7. When did (NAME(S)) take the anti-malarial medicine?**					
Same day after fever	92	44	74	210	80.2%
Next day after fever	13	3	8	24	9.2%
Two day after fever	0	0	0	0	0.0%
Three or more day after fever	1	0	8	9	3.4%
Don't know	1	0	1	2	0.8%
Don't answer	1	0	16	17	6.5%
**8. Are the antimalarial drugs FREE which you and your family took?**					
Yes, they are free for all	38	12	20	70	26.7%
No, only for children, pregnant women and breast-feeding women	2	4	3	9	3.4%
None is FREE	67	31	71	169	64.5%
Don't answer	1	0	13	14	5.3%
**9. When you and your family were infected with malaria, did you take the full dosage of antimalarial drugs which was prescribed by doctor/health**					
**worker?**					
Yes, take full dosages every time	93	35	81	209	79.8%
No, sometimes some dosage was left	15	8	10	33	12.6%
No, some dosages were left every time	0	3	1	4	1.5%
Don't answer	0	1	15	16	6.1%
**9.1 If not all drugs taken, please explain why:**					
We got recovery before finishing all of them	15	9	9	33	89.2%
The drugs made me have headache, nausea	0	0	0	0	0.0%
I am too busy to remember taking all the dosage	0	1	1	2	5.4%
Others: specify	0	1	1	2	5.4%
Don't answer	0	0	0	0	0.0%

* *There were two respondents reported taking both Sp/Fansidar and Artesunate + Lumafantrine*.

# *There were six respondents in Grafton and three respondents in Kossoh reported taking both Artesunate + Lumafantrine and Artesunate + Amodiaquine*.

& *There were three respondents reported taking both Artesunate + Lumafantrine and other*.

@ *There was one respondent reported taking both Artesunate + Lumafantrine and Artesunate + Amodiaquine and other*.

$ *There were two respondents reported taking both Artesunate + Lumafantrine and Amodiaquine*.

% *There was one respondent reported taking both Artesunate + Lumafantrine and Quinine*.

Φ *There was one respondent reported taking both Amodiaquine and other*.

As recalled by the respondents, the health care worker or doctor performed a malaria test when they visited because they suspected that they had contracted malaria (80.2%, 210/262) (χ^2^ = 15.854, *P* = 0.003), and the malaria rapid diagnostic tests (RDT) was the most commonly used test (54.6%, 143/262); however, approximately half of the respondents (55.0%, 144/262) reported that the doctors or health care workers did not explain the test to them (Table 3).

Most respondents took anti-malarial medicine every time (41.2%, 108/262) or almost every time (35.9%, 94/262) they were diagnosed with malaria, and ~79.8% (209/262) of them took the full course of treatment every time as prescribed by the doctor or health care worker. Artesunate and lumefantrine (57.6%, 151/262) and artesunate and amodiaquine (20.6%, 54/262) were the top two choices of combination therapy. The most common reason given for the early termination of a course of treatment was having recovered prior to finishing all the doses (89.2%, 33/37). Moreover, ~80.2% (210/262) of the respondents reported starting anti-malarial medicine on the same day that they noted the fever, and ~64.5% (169/262) of them responded that the anti-malarial medicine were not free (Table 3).

### Malaria Prevention Measures

In this survey, 188 households had long-lasting insecticidal nets (LLINs) (χ^2^ = 2.282, *P* = 0.340), but more nets were required because 189 respondents said that they did not have enough LLINs in their households (*P* = 0.102, Fisher's exact test), so resulting in children under the age of 5 years in 66 households not having LLINs to sleep under (*P* = 0.120, Fisher's exact test). Only 146 respondents had slept under nets the night before the survey (*P* = 0.073, Fisher's exact test). Furthermore, some members in 162 households with nets did not sleep under LLINs (χ^2^ = 18.179, *P* = 0.001), which could lead to cross-infection. Half of the LLINs were distributed by the governmental hospital/health centers (49.6%, 130/262) and community health centers (15.3%, 40/262) (Table 4).

**Table 4:** Malaria prevention practices in the three communities in the Western Area Rural District, Sierra Leone, 2018.

**Question and answer**	**Community**			**Total**	**Percentage**
	**Grafton**	**Jui**	**Kossoh**		
**1. Do you have a mosquito treated bed nets in your home?**					
Yes	79	37	72	188	71.8%
No	29	10	35	74	28.2%
**2. Did you sleep under a mosquito net last night?**					
Yes	66	25	55	146	55.7%
No	36	19	51	106	40.5%
Don't answer	6	3	1	10	3.8%
**3. Is there anyone in your household who did not sleep under treated bed nets last night?**					
Yes	69	37	56	162	61.8%
No	29	7	48	84	32.1%
Don't answer	10	3	3	16	6.1%
**4. Are there enough treated bed nets in your household?**					
Yes	25	8	32	65	24.8%
No	73	39	73	189	72.1%
Don't answer	6	0	2	8	3.1%
**5. Do all children under 5 years in your household sleep under treated bed nets at night?**					
Yes	39	25	38	102	38.9%
No	26	13	27	66	25.2%
No children under 5	41	8	41	90	34.4%
Don't answer	2	1	1	4	1.5%
**6. Where did you get the treated bed nets?**					
Government Hospital/Health Center	58	32^*^	40^#^	130	49.6%
Mobile Clinic	1	0	0	1	0.4%
Community Health Center	5	8	27	40	15.3%
Private Hospital/Clinic	0	7^*^	1	8	3.1%
Pharmacy	0	1	0	1	0.4%
Shop	1	1	8^#^	10	3.8%
Traditional Healer	0	0	0	0	0.0%
Others	24	2	0	26	9.9%
Don't answer	19	2	32	53	20.2%
**7. Did you spray insecticide to kill mosquitoes in your house?**					
Often	9	8	16	33	12.6%
Sometimes	12	13	12	37	14.1%
Seldom	15	9	24	48	18.3%
Never	72	17	54	143	54.6%
Don't answer	0	0	1	1	0.4%
**8. Did you spray insecticide to kill mosquitoes outside your house?**					
Often	0	1	4	5	1.9%
Sometimes	2	2	2	6	2.3%
Seldom	6	5	9	20	7.6%
Never	100	38	87	225	85.9%
Don't answer	0	1	5	6	2.3%
**9. Does your house have doors or windows screen which could stop the mosquitoes to go into the house?**					
Yes	6	14	38	58	22.1%
No	102	33	67	202	77.1%
Don't answer	0	0	2	2	0.8%
**10. Do you and your family often stay outside of the house at night (such as for walk, exercises, enjoy the cool air, work, etc)**					
Often	57	41	86	184	70.2%
Sometimes	38	4	9	51	19.5%
Seldom	8	1	6	15	5.7%
Never	4	1	4	9	3.4%
Don't answer	1	0	2	3	1.1%

* *There were six respondents reported taking the treated bed nets from Government Hospital/Health Center and Private Hospital/Clinic both*.

# *There was one respondent reported taking the treated bed nets from Government Hospital/Health Center and shop both*.

In addition, a total of 143 households never sprayed insecticide indoors to kill mosquitoes (χ^2^ = 19.945, *P* = 0.007), and 225 households never sprayed insecticide outside (*P* = 0.073, Fisher's exact test). Furthermore, more than three quarters of the respondents (77.1%, 202/262) reported that they did not have a door or window screen to prevent mosquitoes from entering their houses (*P* < 0.001, Fisher's exact test). Additionally, most families (89.7%, 235/262) reported staying outside of the house at night (*P* < 0.001, Fisher's exact test) (Table 4).

### Knowledge of Malaria

In this survey, almost all the respondents had heard about malaria (98.1%, 257/262) (*P* = 0.214, Fisher's exact test) and knew that the main route of transmission was through mosquito biting (86.6%, 227/262) (χ^2^ = 5.045, *P* = 0.081). Fever (44.3%, 116/262) (χ^2^ = 4.845, *P* = 0.092), body aches or joint pain (38.9%, 102/262) (χ^2^ = 1.189, *P* = 0.566), and loss of appetite (36.6%, 96/262) (χ^2^ = 2.928, *P* = 0.237) were the top three clinical manifestations they reported. A total of 173 respondents reported that they would always go to see a doctor or health care worker when they suspected they had malaria (χ^2^ = 1.323, *P* = 0.530), while 48 interviewees said they would take some of the anti-malarial medicine kept in their houses first (χ^2^ = 15.322, *P* < 0.001). The expensive costs (mentioned by 176 respondents) was the main factor preventing people from visiting the doctor or health care worker (χ^2^ = 0.059, *P* = 0.969). Sleeping under LLINs (198) (χ^2^ = 1.432, *P* = 0.499) and keeping their surrounding clean (84) (χ^2^ = 5.982, *P* = 0.051) were the most common methods of malaria prevention employed (Table 5).

**Table 5:** Knowledge of malaria transmission, symptom and prevention in the three communities in the Western Area Rural District, Sierra Leone, 2018.

**Question and answer**	**Community**			**Total**	**Percentage**
	**Grafton**	**Jui**	**Kossoh**		
**1. Have you or any member of your family ever heard of an illness called malaria?**					
Yes	107	47	103	257	98.1%
No	1	0	4	5	1.9%
2. In your opinion, what cause malaria?^*^					
Mosquito bites	89	45	93	227	86.6%
Eating immature sugarcane	0	0	0	0	0.0%
Eating dirty food	6	0	1	7	2.7%
Drinking beer/palm Wine	0	0	0	0	0.0%
Drinking dirty water	11	1	4	16	6.1%
Getting soaked with rain	0	0	0	0	0.0%
Cold or changing weather	1	0	1	2	0.8%
Witchcraft I. injections/drugs	0	0	1	1	0.4%
Eating oranges or mangos	0	1	1	2	0.8%
Eating plenty oil	2	1	0	3	1.1%
Sharing razors/blades	1	0	0	1	0.4%
Don't answer	9	2	10	21	8.0%
3. Can you tell any symptoms of malaria?^*^					
Fever	48	27	41	116	44.3%
Excessive sweating	11	2	6	19	7.3%
Feeling cold	36	7	29	72	27.5%
Headache	33	11	11	55	21.0%
Nausea and vomiting	11	10	10	31	11.8%
Dizziness	19	13	34	66	25.2%
Loss of appetite	35	22	39	96	36.6%
Body ache or joint pain	46	16	40	102	38.9%
Body weakness	31	25	32	88	33.6%
Refusing to eat or drink	0	1	1	2	0.8%
Jaundice	0	3	3	6	2.3%
Dark urine	14	1	11	26	9.9%
Others	5	0	5	10	3.8%
Don't answer	0	0	1	1	0.4%
4. What should you do when you suspect getting malaria infection?^*^					
Must go to see the doctor/health worker	67	32	74	173	66.0%
Take some anti-malarial drugs kept in house	26	1	11	48	18.3%
Not necessary to see the doctor/health worker and take drugs	3	0	0	3	1.1%
Go to see the doctor/health worker only when shown severe condition	2	0	13	15	5.7%
Others: Specify.	9	1	8	18	6.9%
Don't answer	1	3	3	7	2.7%
5. What are the reasons you think that someone would not go to see the doctor/health worker when he or she gets malaria infection?^*^					
We are all used to it and no necessary to see the doctor	0	1	1	2	0.8%
Prayers could make us recover	0	0	0	0	0.0%
They are strong enough and could recover even if they do not see the doctor	0	0	0	0	0.0%
They have anti-malarial drugs and take it when necessary	1	11	7	19	7.3%
It is unnecessary to see the doctor/health worker for recovering from malaria	1	1	0	2	0.8%
It is too expensive to see the doctor/health worker	73	32	71	176	67.2%
It is too far to go to see the doctor/health worker	0	1	2	3	1.1%
Other reasons	33	0	21	54	20.6%
Don't answer	4	2	7	13	5.0%
6. How can someone protect themselves against malaria?^*^					
Sleep under treated bed net	78	38	82	198	75.6%
Untreated mosquito net	1	0	0	1	0.4%
Use mosquito repellent	2	1	1	4	1.5%
Use mosquito insecticide spray (Shelltox)	12	5	3	20	7.6%
Take preventive medication	0	9	2	11	4.2%
Indoor residual spray (IRS)	0	3	4	7	2.7%
Use mosquito coils	7	3	5	15	5.7%
Cut grass around house	6	4	1	11	4.2%
Eliminate stagnant water	8	4	9	21	8.0%
Keep surroundings clean	42	9	33	84	32.1%
Don't drink dirty water	1	0	2	3	1.1%
Contaminated food	3	0	4	7	2.7%
Use mosquito screens on windows and doors	0	1	5	6	2.3%
Others: specify	14	0	10	24	9.2%
Don't answer	1	1	1	3	1.1%
**7. Where do you think the mosquito larvae live? DO NOT PROMPT ANSWER**					
In water	61	33	59	153	58.4%
In weed	0	2	2	4	1.5%
In rubbish	24	6	24	54	20.6%
I don't know	19	0	17	36	13.7%
Don't answer	4	6	5	15	5.7%

* *It is a multiple-choice question*.

## Discussion

Malaria remains one of the most serious public health issues and is responsible for high proportions of morbidity and mortality in Sierra Leone. In the present study, overall high levels of knowledge regarding the causes of malaria, prevention mechanisms and symptoms, and active seeking of treatment for malaria from health care providers were found among Sierra Leoneans, similar to the results in the MIS 2013 and 2016 (2, 10); these results with regards to knowledge and practices support malaria control (11–13).

Globally, vector control is the most commonly accomplished through the use of LLINs and indoor residual spraying (IRS), with the aim of reducing the transmission by preventing human–vector contact and killing vector mosquitoes (14, 15). The goal was for the entire at-risk population to employ preventive measures, including the use of LLINs, IRS and larval source management, by 2017 in Sierra Leone. However, 72.1% of the respondents required more nets, which may explain the high percentage (61.8%, 162/262) of households and the high proportion of children under 5 years old who did not sleep under the nets at night according to this survey. This indicates a large gap in obtaining and using nets by the most vulnerable groups who are not benefiting as much as hoped from preventive malaria interventions. Moreover, screens for doors and windows that are cost-effective to install and maintain are a supplementary public health intervention to prevent humans from being bitten by mosquito vectors indoors, thereby significantly reducing the transmission of malaria. However, screens seem to have been neglected in the regular malaria control campaigns (16, 17), resulting in fewer than 25% of households having door or window screen in this survey, and a general low awareness of the role of screens. In addition, a low level of implementation of IRS and outside spraying and a high level of engagement in activities outside of the house at night were found, despite the presence of many mosquitoes. Therefore, how to improve residents' outdoor protection, or reduce outdoor activities at night, as well as to further improve the awareness and correct use of protective measures, can become an important content of the future malaria campaign.

The early diagnosis and prompt treatment of malaria reduces the transmission of the disease and prevents deaths. It is critical for people to seek diagnosis and care as soon as they experience any symptoms of malaria. It is encouraging that more than 80% of the respondents reported that they visited the doctor in a timely manner, and a similar percentage of respondents reported being tested for malaria, usually with RDTs not microscopy, which is the standard method for malaria diagnosis. However, approximately three-quarters of the respondents said that the doctors did not interpret the test results for the patients. Therefore, a good opportunity to provide health education and promote malaria control and prevention is being missed. A home-based RDT that could be performed by trained family members rather than at a healthcare facility may improve the timeliness of the diagnosis of malaria (18–20). Moreover, the reported adherence to completing the course of antimalarial treatment was close to 80% at the clinic or if self-administration at home. Reasons for the lack of adherence were reported to be sickness after the first dose, insufficient food to take the medicine, forgetting to take the medicine, and poor instructions provided by the community health center (CHC) (21). In addition, the cost of medical treatment and the distance from the hospital must also be considered.

However, there were some limitations of this study. First, this was a small-sample cross-sectional study conducted in three communities, and the findings are not yet nationally representative. Second, the collected data were based on respondent recall, and the data may be biased. Therefore, the results of the study highlight the need for more extensive investigations of the KAP regarding malaria in Sierra Leone.

## Conclusion

In conclusion, in these three communities, public awareness of malaria prevention measures was relatively high in this survey, but the malaria burden was still higher than because the limited use of preventive measures against malaria potentially. Thus, the existing knowledge of malaria should be sustained and reinforced, and the availability and use of malaria prevention measures should be promoted to achieve the goals of the SL-NMSP.

## Data Availability Statement

The raw data supporting the conclusions of this article will be made available by the authors, without undue reservation.

## Ethics Statement

The studies involving human participants were reviewed and approved by the Sierra Leone Ethics and Scientific Review Committee. Written informed consent to participate in this study was provided by the participants' legal guardian/next of kin.

## Author Contributions

JY and CZ conceived the study, developed questionnaire, and conducted fieldwork. LW and JY analyzed data and wrote the manuscript. SS and BK contributed to the study design. EN and AK conducted fieldwork and supervised fieldwork. XH, XC, XW, and WL contributed to data collation and cross-checking. All authors read and approved the final draft.

## Conflict of Interest

The authors declare that the research was conducted in the absence of any commercial or financial relationships that could be construed as a potential conflict of interest.
